# Suppression Attack Against Multicast Protocol in Low Power and Lossy Networks: Analysis and Defenses

**DOI:** 10.3390/s18103236

**Published:** 2018-09-26

**Authors:** Cong Pu, Xitong Zhou

**Affiliations:** 1 Weisberg Division of Computer Science, Marshall University, Huntington, WV 25755, USA; 2 MS Graduate of Computer Science, Marshall University, Huntington, WV 25755, USA; zhou34@marshall.edu

**Keywords:** Denial-of-Service attack, low power and lossy networks, multicast protocol, suppression attack

## Abstract

With increasingly prevalent wireless sensors and devices, low power and lossy networks (LLNs) play an essential role in the realization of ubiquitous computing and communication infrastructure, which, in turn, leads to enhanced data accessibility and availability. A multicast protocol for LLNs (MPL), has been standardized to provide both efficient and reliable multicast communication. Due to the shared wireless medium, lack of tamper resistance, and inherent resource constraints, MPL-based LLNs are undoubtedly vulnerable to various Denial-of-Service (DoS) attacks. In this paper, we propose a heuristic-based detection scheme, called HED, against the suppression attack in MPL-based LLNs, where a malicious node multicasts a series of spoof data messages with continuous sequence numbers to prevent normal nodes from accepting valid data messages and cause them to delete cached data messages. In the HED, each node maintains an increment rate of the minimum sequence number in the Seed Set to detect the potential malicious node by comparing the recent increment of sequence numbers with the heuristically calculated increment threshold of sequence numbers. We evaluate the proposed scheme through extensive simulation experiments using OMNeT++ and compare its performance with original MPL with and without adversary, respectively. The simulation results show high detection rate and packet reception rate but low false detection rate, and indicate that the proposed scheme is a potentially viable approach against the suppression attack in MPL-based LLNs.

## 1. Introduction

A rapidly growing number of wireless sensors and devices (later nodes), and hybrid networks are leading the emergence of Internet-of-Things (IoT) and its applications, where a myriad of multiscale nodes are seamlessly blended and communicate with each other [1]. It has been predicted that 11 billion wirelessly connected nodes will be available for IoT applications in 2018, a 33% increase from 2017, and will reach 20.4 billion by 2020 [2]. Economic growth of IoT-based services and applications is also said to be considerable for businesses. It is probable that the whole annual economic impact caused by IoT will be in the range of $2.7–6.2 trillion by 2025 [3]. With the prevalence of WiFi and 4G LTE, cloud computing, social networking, and the recent technological advances in embedded devices and sensor networks, we envision a future in which wireless IP-enabled smart nodes under IoT will enhance data accessibility and availability, and lead to the further improvement of our lives.

As a major part of IoT, low power and lossy networks (LLNs) play an essential role in the realization of ubiquitous computing and communication, where a set of resource-constrained nodes in terms of communication, computation, memory, and energy communicates among themselves directly or indirectly via lossy links. With the increasing demand of sharing information and knowledge and coordinating decisions, the Internet Engineering Task Force (IETF) Working Group has proposed a multicast protocol for LLNs, also referred to as MPL [4], as the multicast communication standard. However, MPL-based LLNs are unquestionably vulnerable to various Denial-of-Service attacks [5] because of the inherent shared wireless medium and the lack of physical protection and security requirements of network protocol. It has been noted that DoS attacks primarily target service availability to diminish the network capability by disrupting network protocol or interfering with any on-the-fly communication, rather than subverting the service itself. To address these issues, the MPL standard [4] makes the recommendation to employ the use of link-layer security mechanisms (e.g., IEEE 802.15.4 AES-128 [6] and Cisco’s CG-Mesh [7]) to prevent an outside attacker who has no access to cryptographic materials from injecting spoof messages. However, the link-layer security mechanisms are incapable of countering an inside attacker who can capture and compromise a legitimate node, gain access to all stored information (e.g., public and private keys), and reprogram it to behave maliciously [8]. The current MPL implementations often do not deploy extra security operations that can significantly consume computing power and affect the performance of resource-constrained nodes [5,9,10]. In addition, a security threat analysis of LLNs presented in [11] is limited to discussing only well-known attacks with fundamental countermeasures. Thus, MPL-based LLNs are open to new attack wherein a malicious node can easily multicast a series of spoof data messages to disrupt routing protocol and interfere with on-going communications.

In light of the above, we investigate a suppression attack and propose a heuristic-based detection scheme, called HED, to efficiently mitigate the suppression attack in MPL-based LLNs. In the suppression attack, a malicious node multicasts a series of spoof data messages with continuous sequence numbers to prevent legitimate nodes from accepting valid data messages, and cause them to delete their cached data messages as well. Although the countering of jamming attacks and its variants have been extensively studied [12], the suppression attack and its countermeasure in MPL-based LLNs are under-explored and remain in their infancy. Our major contributions are summarized in the following:

- We significantly extend our previous work [13], and analyze the suppression attack with a preliminary result in MPL-based LLNs. This is the first in-depth work that investigates the performance impact of suppression attack in MPL-based LLNs.
- We propose a heuristic-based detection scheme, called HED, to efficiently mitigate the suppression attack in MPL-based LLNs. In the HED, each node maintains an increment rate of the minimum sequence number in the Seed Set, and compares the recent increment of sequence numbers within a time period with the heuristically calculated increment threshold of sequence numbers to detect potential malicious node.
- We propose a simple analytical model of the HED and show its numerical result in terms of miss detection rate. We also revisit and implement the original MPL with and without adversary for performance comparison. In addition, the original MPL without adversary will be used as the upper bound of packet reception rate.

We develop a customized discrete event-driven simulation framework by using OMNeT++ [14] and evaluate its performance through extensive simulation experiments in terms of detection rate, packet reception rate, false detection rate, and changes of increment rate. The simulation results indicate that the proposed countermeasure is a viable detection approach to suppression attack in MPL-based LLNs.

The remainder of the paper is organized as follows. Prior approaches are presented and analyzed in Section 2. The basic MPL operations, analysis of suppression attack with a preliminary result, and the proposed countermeasure are presented in Section 3. An analytical model of the proposed countermeasure is presented in Section 4. Performance evaluation, including extensive simulation experiments and analysis, is provided in Section 5. In Section 6, we analyze and compare the suppression attack with well-known jamming attack in terms of attack method, stealthiness, attack energy efficiency, and level of denial of service. Finally, Section 7 concludes the paper with possible future research directions.

## 2. Related Work

In this section, we categorize and analyze a variety of existing attacks and countermeasures in terms of wireless ad hoc networks, low power and lossy networks, and Internet of Things.

### 2.1. Wireless Ad Hoc Networks

In [15], a camouflage-based detection scheme, called CAM, is proposed to detect the forwarding misbehavior in energy harvesting motivated networks (EHNets). The basic idea is that each node hides its current operational status and pretends not to overhear or monitor any on-going forwarding operation of its adjacent nodes to detect a deep lurking malicious node. A cooperative countermeasure (EYES) [16] is an extended version of the CAM, where each node periodically requests its adjacent nodes of a limited history of forwarding operations, and validates any prior uncertain forwarding operation to detect the forwarding misbehavior. The AAA [17] is proposed to detect the stealthy collision attack in EHNets, where each node forwards a data packet, and then monitors the subsequent packet transmission of its one-hop downstream node and waits for an explicit acknowledgment packet from its two-hop downstream node. In the SCAD [18], a single checkpoint-assisted approach integrated with timeout and hop-by-hop retransmission techniques is proposed to detect the selective forwarding attack in wireless sensor networks (WSNs), where single or multiple malicious nodes randomly or selectively drop any incoming packet. In [19], a novel reactive routing scheme integrated with bypass technique is proposed to mitigate the selective forwarding attack in WSNs, where each node estimates and observes the parent node’s reliability and link quality, and then decides whether to forward the packet.

In [20], a DSR-based bait detection scheme incorporated with a digital signature technique is proposed to detect routing misbehaviors in mobile ad hoc networks (MANETs), where a source node broadcasts a route request packet with a fictitious destination node to lure potential malicious nodes to reply a fake route reply packet. Yang et al. [21] proposed a polynomial-based compromise-resilient en-route filtering (PCREF) scheme against false data injection attack in cyber-physical networked systems. The PCREF is designed by adopting message authentication polynomials rather than message authentication codes and clusters to avoid utilizing node locations. Zou et al. [22] examined security vulnerabilities and threats imposed by the inherent open nature of wireless communications and presents a variety of efficient defense mechanisms for improving the wireless network security among different layers.

### 2.2. Low Power and Lossy Networks

Over the last few years, researchers have explicitly studied the numerous security issues associated with LLNs. In the VeRA [23], a version number and rank authentication security scheme based on one-way hash chains are proposed to secure the IPv6 routing protocol (RPL) [24] in LLN, where the misbehaving nodes illegitimately increase the version number of directed acyclic graph information object (DIO) message and compromise illegal rank values. To protect against the attackers that send DIO messages with higher version number values or that publish a high rank value, the version numbers are bound with authentication data and signature. In [25], a rank attack that aims at the rank property in RPL and its performance impact are investigated in WSNs, where the adversary can compromise the rank rule to downgrade the RPL performance. Four adversarial scenarios motivated by violating rank rule permanently and non-permanently and their potential performance impact are analyzed. In the Dodge-Jam [26], a lightweight anti-jamming technique suitable for LLN environments is proposed to address the stealthy jamming attacks with small overhead. Perazzo et al. [27] investigated the DODAG Information Object suppression attack, which can severely degrade the routing service in RPL. The CMD [28] proposes a monitor-based approach to mitigate the forwarding misbehaviors in LLNs, where each node monitors the forwarding behaviors of the preferred parent node to observe the packet loss rate, compares the observation result with the collected packet loss rate from one-hop neighbor nodes, and detects the forwarding misbehaviors of the preferred parent node. In [29], a dynamic threshold mechanism is proposed to mitigate the destination advertisement object (DAO) inconsistency attack in RPL-based LLNs, where a malicious node intentionally drops the received data packet and replies the forwarding error packet to cause the parent node to discard valid downward routes in the routing table. The [30] identified and investigated a new type of Denial-of-Service attack, called hatchetman attack, in LLNs, where a malicious node manipulates the source route header of the received packet, and then generates and sends the invalid packets with error route to legitimate nodes.

### 2.3. Internet of Things

The SVELTE [31] proposes a novel intrusion detection system to secure Low-Power Wireless Personal Area Network (6LoWPAN) from the network layer and routing attacks. Beigi-Mohammadi et al. [32] designed and implemented an intrusion detection system that can be modified to employ RPL routing protocol in neighborhood area network. Hummen et al. [33] proposed two complementary and lightweight defense mechanisms to counter fragmentation attack in the adaptation layer of 6LoWPAN. The security capability of IEEE 802.15.4 MAC protocol as well as the limitations thereof in the context of IoT are analyzed in [34]. A security threat analysis of RPL has been performed in [11], where potential security issues and fundamental countermeasures are presented. A more detailed survey of DoS attacks in IoT can be found in [35,36]. In [5], the history of research efforts in RPL-based LLNs and future research directions on which LLNs should evolve have been reviewed and discussed.

In summary, various attacks and their countermeasures have been well studied throughout various networks and environments. However, little attention has been shown to the suppression attack in the realm of MPL-based LLNs.

## 3. Countermeasure to Suppression Attack

In this section, we begin with an overview of the multicast protocol for low power and lossy networks. Then, we analyze the suppression attack with a preliminary result. Finally, we present both system and adversary models and propose a heuristic-based detection scheme, also called HED, against the suppression attack in MPL-based LLNs.

### 3.1. Overview of Multicast Protocol

Multicast protocol for low power and lossy networks, also referred to as MPL [4], is a IPv6 multicast forwarding protocol in resource-constrained networks. The MPL disseminates messages to all the nodes within the same MPL domain without the need of constructing or maintaining any multicast forwarding topology. To exchange control-plane and data-plane messages in a highly robust, energy efficient, and scalable manner, the MPL relies on the Trickle Algorithm [37]. The basic idea of Trickle Algorithm is to optimize the message transmission frequency based on network conditions. Specifically, the frequency is increased whenever an inconsistent network information is received, and decreased in the opposite case.

When a source node has a data message to send within an MPL domain, it generates a message that includes the MPL domain address as a destination address. Here, the MPL domain is a scope zone in which nodes subscribe to the same MPL domain address and also participate in the dissemination of MPL data and control messages. The source node also piggybacks its identifier, a newly generated sequence number, and the payload in the data message. It then schedules a multicast of data message using the Trickle Algorithm, which is completed without any prior indication that the neighboring nodes have received the data message. After transmitting the data message a limited number of times, the source node terminates the transmission process of data message.

**Figure 1:**
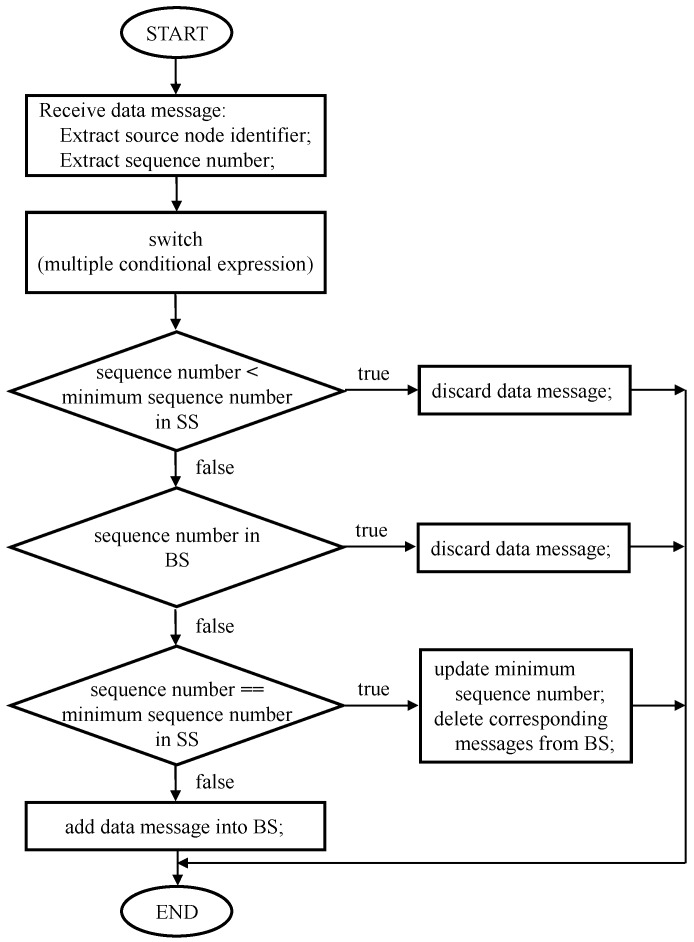
An information flow of a normal node that receives a data message. Here, *SS* and *BS* denote Seed Set and Buffered Message Set, respectively.

When the node that subscribes to the same MPL domain address has received the data message, it extracts the source node identifier and sequence number to determine whether or not the data message has been received. If the sequence number is less than the minimum sequence number maintained in the Seed Set, or equal to the sequence number stored in the Buffered Message Set, the receiving node discards the data message because it believes that it has received the data message before. In the MPL, a Seed Set records a sliding window that is used to determine the sequence number of data message that the node is willing to receive. The Seed Set consists of three components: the identifier of source node, the minimum sequence number that the node is willing to receive, and the lifetime of the Seed Set entry. Additionally, a Buffered Message Set records recently received data messages that have a sequence number greater than the minimum sequence number in the Seed Set. The Buffered Message Set is composed of three components: the identifier of source node, the sequence number of data message, and the payload of data message. If the sequence number is equal to the minimum sequence number in the Seed Set, the receiving node updates the minimum sequence number to one greater than the received data message’s sequence number. At this point, it deletes any data message that has a sequence number less than the updated minimum sequence number from the Buffered Message Set. If the sequence number is greater than the minimum sequence number in the Seed Set, but not stored in the Buffered Message Set, the receiving node adds the received data message into the Buffered Message Set. Then, the receiving node multicasts the received data message using the Trickle Algorithm to all neighbor nodes that subscribe to the same MPL domain. Here, an overall information flow of normal node that receives a data message is shown in Figure 1.

Nodes that subscribe to the same MPL domain also periodically exchange a MPL control message using the Trickle Algorithm to discover new data messages that have not been received, where MPL control message contains the information of Seed Set and Buffered Message Set. If a node discovers a neighbor node that has not received certain data messages, it multicasts those data messages using the Trickle Algorithm.

### 3.2. Analysis of Suppression Attack

The MPL uses a sequence number to normally maintain the order of data messages transmitted from a source node. However, the sequence number can be misused by an adversary to attack the network. For example, a malicious node can intentionally multicast a series of spoof data messages with continuous sequence numbers within a short period of time to increase the minimum sequence number stored in the Seed Set. This can prevent the legitimate nodes from accepting valid data messages with a sequence number less than the minimum sequence number from the source node, and cause them to delete data messages with a sequence number less than the minimum sequence number from the Buffered Message Set.

**Figure 2:**
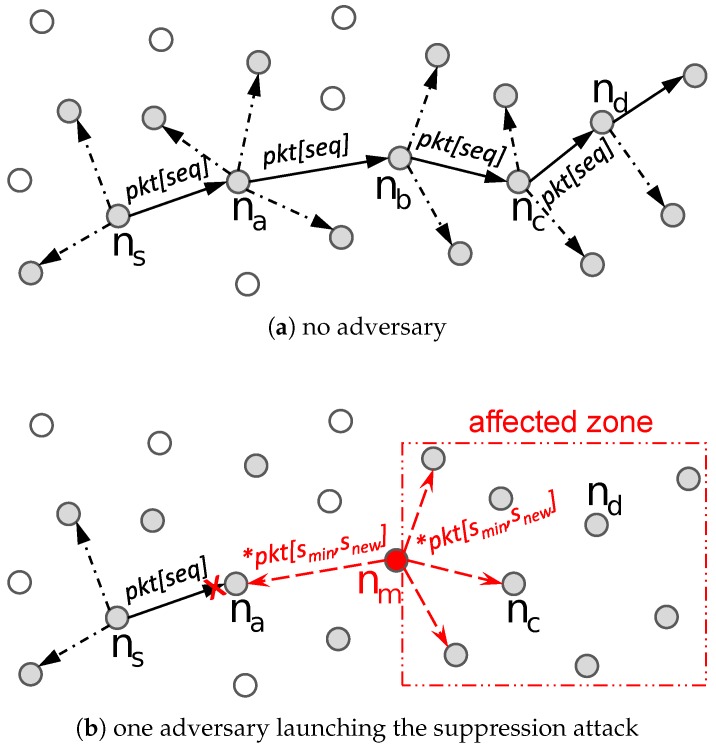
An example of multicasting a data message: (**a**) no adversary; and (**b**) one adversary launching the suppression attack.

Suppose that a source node ($n_{s}$) multicasts a data message (pkt[seq]) with a sequence number (*seq*) to a node ($n_{d}$) via intermediate nodes (e.g., $n_{a}$, $n_{b}$, and $n_{c}$), as shown in Figure 2a, where other nodes that subscribe to the same MPL domain also could receive and multicast the data message. We implicitly assume that each node faithfully and collaboratively multicasts pkt[seq] and thus, $n_{d}$ can successfully receive the data message. However, a malicious node can easily launch the suppression attack by multicasting a series of spoof data messages with continuous sequence numbers to increase the minimum sequence number stored in the Seed Set at legitimate nodes. In Figure 2b, a malicious node ($n_{m}$) multicasts a series of spoof data messages with continuous sequence numbers ranging between $s_{min}$ and $s_{new}$ within a short period of time, denoted as ${}^{*}pkt\left[s_{min},s_{new}\right]$, to its neighbor nodes. The malicious node could increase the time of multicasting a series of spoof data messages with continuous sequence numbers, however, the malicious node does not benefit from doing so because the minimum sequence number maintained in the Seed Set will not be increased greatly. Here, $s_{min}$ is the minimum sequence number stored in the Seed Set at neighbor nodes. Through frequently exchanged MPL control messages, it is not hard for a malicious node to find the stored minimum sequence number at neighbor nodes. When a legitimate node, e.g., $n_{a}$, receives ${}^{*}pkt\left[s_{min},s_{new}\right]$ from $n_{m}$, it updates the minimum sequence number stored in the Seed Set to ($s_{new}+1$) and then deletes any data message that has sequence number less than ($s_{new}$ + 1) from the Buffered Message Set. When the source node ($n_{s}$) generates and multicasts a new data message (pkt[seq]), where seq < ($s_{new}$ + 1), the legitimate node ($n_{a}$) will not accept pkt[seq] based on the MPL protocol since pkt[seq] has the sequence number less than the minimum sequence number stored in the Seed Set. As shown in Figure 2b, due to the suppression attack, a great number of legitimate nodes that are located in the affected zone cannot accept valid data messages from the source node, suffering from denial of service.

**Figure 3:**
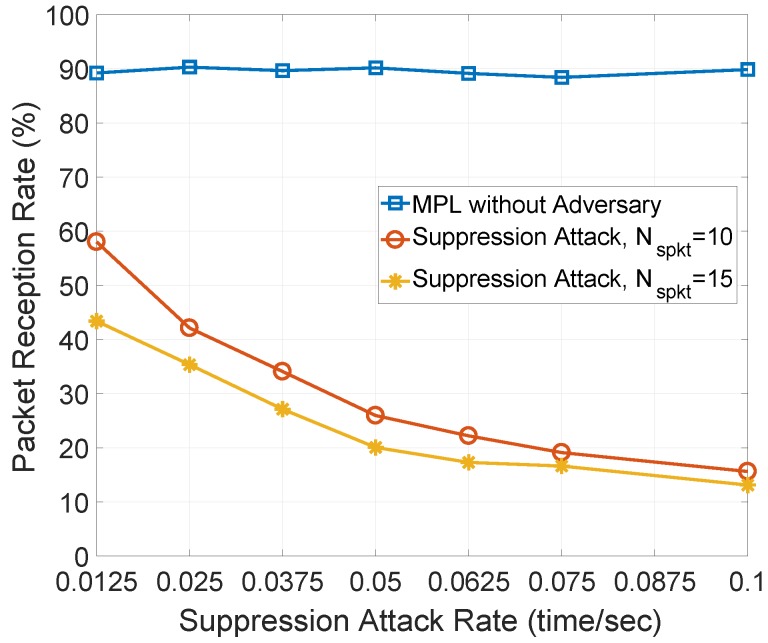
The packet reception rate against suppression attack rate and number of spoof data messages ($N_{spkt}$). Here, we consider a network area (150 × 150 (m^2^)), where 51 nodes including one source node and five malicious nodes are uniformly distributed.

In Figure 3, we measure the packet reception rate against the suppression attack rate and number of spoof data messages ($N_{spkt}$). In this paper, the suppression attack rate indicates how frequently a malicious node multicasts a series of spoof data messages with continuous sequence numbers to increase the minimum sequence number stored in the Seed Set. As shown in Figure 3, when the suppression attack rate increases, the packet reception rate of legitimate node decreases quickly. This is because the series of spoof data messages with continuous sequence numbers makes the minimum sequence number increase, and data messages with smaller sequence number from source node will not be accepted. As the number of spoof data messages $N_{spkt}$ increases in each attack, a lower packet reception rate is observed compared to that of $N_{spkt}=10$. In effect, additional spoof data messages can make the minimum sequence number increase greatly and quickly, and more data messages from source node will be rejected, which leads to lower packet reception rate. In summary, the suppression attack can be easily launched to prevent a great number of legitimate nodes that subscribe to the same MPL domain from receiving valid data messages, and finally leads to denial of service in MPL-based LLNs.

### 3.3. HED: Heuristic-Based Detection Scheme

**System and Adversary Models:** A low power and lossy network running with MPL is considered, where a set of resource-constrained nodes including single source node communicates among themselves directly or indirectly through lossy links. Each node is uniquely identified by an identifier, e.g., an IPv6 address. Due to the shared wireless medium, lack of tamper resistance, and inherent resource constraints, we assume that an adversary is able to capture and compromise a legitimate node, gain access to all stored information (e.g., public and private keys), and reprogram it to behave maliciously [8]. However, we do not consider node capture attack [38], where an adversary can capture a legitimate node from the network as the first step to further conduct different types of attacks. The primary goal of the adversary is to disrupt the MPL and interfere with any on-going communication. A malicious node will not intentionally drop all received multicast messages (i.e., blackhole attack) because the legitimate nodes could still receive the messages from other multicasting nodes. The malicious node may inject bogus messages into the network to consume the scarce network resource (i.e., bogus data injection attack), but this attack can be easily prevented by using the technique proposed in [39]. In this paper, we primarily focus on the multicasting misbehavior and its corresponding adversarial scenario, where the malicious node multicasts a series of spoof data messages with continuous sequence numbers to suppress normal nodes to accept valid data messages and cause them to delete their cached data messages. We only deal with the scenario that the malicious node acts alone, and the problem of colluding malicious nodes is out of the scope of this paper. Note that we do not consider suppression attack combined with other general attacks, such as sybil, collision or jamming, wormhole, or vampire attacks.

**Major Operations:** The basic idea of HED is that each node maintains an increment rate of the minimum sequence number in the Seed Set, and compares the recent increment of sequence numbers within a time period with the heuristically calculated increment threshold of sequence numbers to detect the potential malicious node in MPL-based LLNs. The HED has three major operations. First, each node records a trace of multicast operations of neighbor nodes executed during an observation window (ω), and maintains a multicast trace table (*MT*) to monitor their multicast operations. We deploy an observation window (ω) to detect anomalous increment of sequence numbers within a time period, and ω is adaptively adjusted based on the number of detected multicasting misbehaviors of suspected malicious node. Here, ω is a system parameter and its impacts on the performance are observed in Section 5. The multicast trace table consists of five components: neighbor node’s id (*nid*), sequence number of the first received data message within observation window (*fs*), timestamp of the first received data message within observation window ($t_{fp}$), sequence number of the last received data message within observation window (*ls*), and timestamp of the last received data message within observation window ($t_{lp}$). Here, each entry in multicast trace table brings only an extra 12 bytes of overhead in the memory, where 4 bytes are for node’s id and 2 bytes are for every other four components. For example, as shown in Figure 2b, suppose a malicious node ($n_{m}$) multicasts a series of spoof data messages with continuous sequence numbers in range of $s_{min}$ to $s_{new}$ within a time period between $t_{begin}$ and $t_{end}$, denoted by ${}^{*}pkt\left[s_{min},s_{new}\right]$, to its neighbor nodes. When a legitimate node (e.g., $n_{a}$) receives ${}^{*}pkt\left[s_{min},s_{new}\right]$, it updates the corresponding entry in the *MT*, $MT_{a}\left[m\right].fs=s_{min}$, $MT_{a}\left[m\right].t_{fp}=t_{begin}$, $MT_{a}\left[m\right].ls=s_{new}$, and $MT_{a}\left[m\right].t_{lp}=t_{end}$. In this example, we implicitly assume that the time period of multicast operations, ($t_{end}-t_{begin}$), is within the observation window ω. However, our approach is not dependent on this assumption and ($t_{end}-t_{begin}$) is not required to be within ω.

Second, we modify the Seed Set (*SS*) and introduce an additional component: increment rate of the minimum sequence number within observation window. Thus, the Seed Set *SS* consists of four components: identifier of source node (*nid*), minimum sequence number that the node is willing to receive ($s_{min}$), lifetime of the Seed Set entry ($t_{life}$), and increment rate of the minimum sequence number within observation window ($R_{inc}$). Here, the $R_{inc}$ in Seed Set brings only an extra 2 bytes of overhead in the memory. $R_{inc}$ indicates how much the minimum sequence number has increased per second, and it is updated by the low pass filter with a filter gain constant α,

(1) $$R_{inc}=\alpha \cdot R_{inc}+\left(1-\alpha \right)\cdot R_{rec}^{k}.$$

The basic idea of Equation (1) is that $R_{inc}$ is calculated with the currently observed increment rate of the minimum sequence number within observation window and historical statistics of increment rate of the minimum sequence number. Here, a historical statistics of increment rate of the minimum sequence number is represented by $R_{rec}^{k}$, which is the most recently calculated increment rate of minimum sequence number, and it can be expressed as,

(2) $$R_{rec}^{k}=\frac{ls^{k}-fs^{k}}{t_{lp}^{k}-t_{fp}^{k}}.$$

The basic idea of Equation (2) is to divide the increment of sequence number ($ls^{k}-fs^{k}$) by the elapsed time period ($t_{lp}^{k}-t_{fp}^{k}$) within $k^{th}$ observation window. Thus, the increment rate of the minimum sequence number $R_{inc}$ can be expressed as,

(3) $$R_{inc}=\alpha \cdot R_{inc}+\left(1-\alpha \right)\cdot \frac{ls^{k}-fs^{k}}{t_{lp}^{k}-t_{fp}^{k}}.$$

Here, α is a system parameter and its changes (α ∈ [0.2, 0.8]) and impacts on the increment rate $R_{inc}$ are also observed in Section 5.

Third, at the end of each observation window, each node examines its multicast trace table with an increment rate of the minimum sequence number in the Seed Set to detect any anomalous increment of sequence numbers that was caused by potential multicasting misbehaviors. If the recent increment of sequence numbers within observation window is larger than the heuristically calculated increment threshold of sequence numbers, the corresponding multicast operations are suspected as a multicasting misbehavior and the number of detected multicasting misbehaviors ($c_{mis}$) of suspected node is increased by one. Additionally, the observation window of the suspected node is reduced by half, $\frac{\omega }{2}$. When the $c_{mis}$ reaches a threshold value φ, the node broadcasts an *Isolate* packet to its all one-hop neighbor nodes to prevent neighbor nodes from accepting any message from the suspected node.

**Figure 4:**
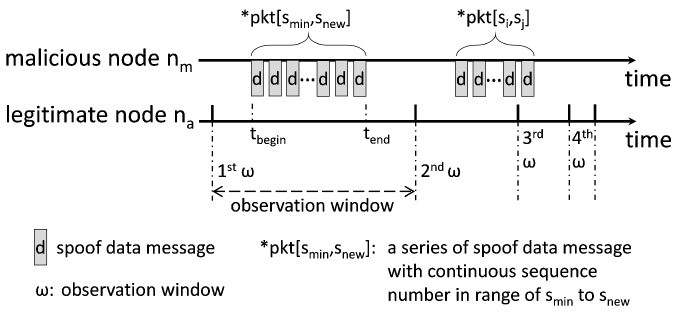
Example of adaptively adjusted observation window ω based on the detected multicasting misbehavior.

**Figure 5:**
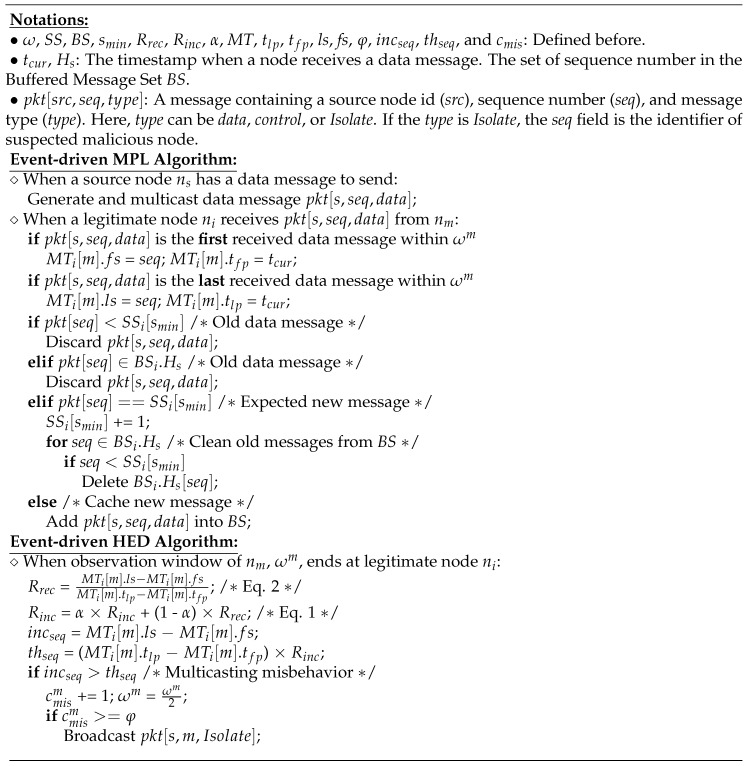
The pseudo code of MPL protocol and the proposed HED scheme.

In Figure 4, for example, a malicious node ($n_{m}$) multicasts a series of spoof data messages with continuous sequence numbers ${}^{*}pkt\left[s_{min},s_{new}\right]$ within a time period between $t_{begin}$ and $t_{end}$ to a legitimate node ($n_{a}$). At the end of first ω, $n_{a}$ observes the actual increment of sequence numbers based on its multicast trace table, $inc_{seq}=\left(MT_{a}\left[m\right].ls-MT_{a}\left[m\right].fs\right)$, calculates the most recent increment rate of sequence numbers based on Equation (2), updates increment rate of the minimum sequence number based on Equation (3), and then heuristically calculates the increment threshold of sequence numbers, $th_{seq}=(MT_{a}\left[m\right].t_{lp}-MT_{a}\left[m\right].t_{fp})\times R_{inc}$. If $inc_{seq}>th_{seq}$, the multicast operations of $n_{m}$ are suspected as the multicasting misbehavior, the number of detected multicasting misbehaviors of $n_{m}$, $c_{mis}^{m}$, is increased by one, and the observation window of $n_{m}$, $\omega^{m}$, is reduced by half, $\frac{\omega^{m}}{2}$. Additionally, the observation window of suspected node becomes shorter, and the multicast operations of a malicious node can be observed more often, and can result in more detections of multicasting misbehaviors. Thus, the smaller the observation window is, the more often the multicasting misbehaviors of malicious node can be detected. Both major operations of MPL protocol and HED scheme are summarized in Figure 5.

## 4. Analysis of the Proposed Countermeasure

In this section, we analyze the HED in terms of average miss detection rate. When multiple spoof data messages are frequently lost because of the bad channel quality, however, a series of spoof data messages may not cause the increment of sequence numbers to be larger than the heuristically calculated increment threshold of sequence numbers, resulting in miss detection. In Figure 4, a malicious node $n_{m}$ multicasts a series of spoof data messages with continuous sequence numbers ${}^{*}pkt\left[s_{min},s_{new}\right]$ within a time period between $t_{begin}$ and $t_{end}$ to a legitimate node $n_{a}$. Due to the bad channel quality, multiple spoof data messages can be lost during the transmission from $n_{m}$ to $n_{a}$. Then, $n_{a}$ may observe an increment of sequence numbers to be smaller than the heuristically calculated increment threshold, which results in a miss detection. In this analysis, we assume that the bad channel quality in terms of channel error primarily causes packet loss, and the channel error rate ($r_{cer}$) is set to 10%.

Let $P_{miss}$ be the average miss detection rate observed in the observation window, which can be expressed as

(4) $$P_{miss}=1-\sum_{i=0}^{N_{atk}}P_{det}^{i}\cdot P_{atk}^{i}$$

where

(5) Patki=iNatk·Ratki·(1−Ratk)Natk−i

(6) Pdeti=∑Nlst=0Nspfi−thseqNlstNspfi·rcerNlst·(1−rcer)Nspfi−Nlst

(7) $$N_{spf}^{i}=N_{spkt}\cdot i$$

Here, $N_{atk}$ is the total number of times that the malicious node decides whether to perform suppression attack and broadcast a series of spoof data messages, $R_{atk}$ is the suppression attack rate, and $N_{lst}$ is the number of spoof data messages that get lost during transmission due to bad channel quality, $N_{spkt}$ is the number of spoof data messages broadcasted by malicious node in each attack, and $th_{seq}$ is the heuristically calculated increment threshold of sequence numbers. $N_{spf}^{i}$ is the number of spoof data messages broadcasted by malicious node during the *i* number of suppression attacks. $P_{atk}^{i}$ is the probability that the malicious node performs the *i* number of suppression attacks during the observation window. $P_{det}^{i}$ is the detection rate of the *i* number of suppression attacks.

**Figure 6:**
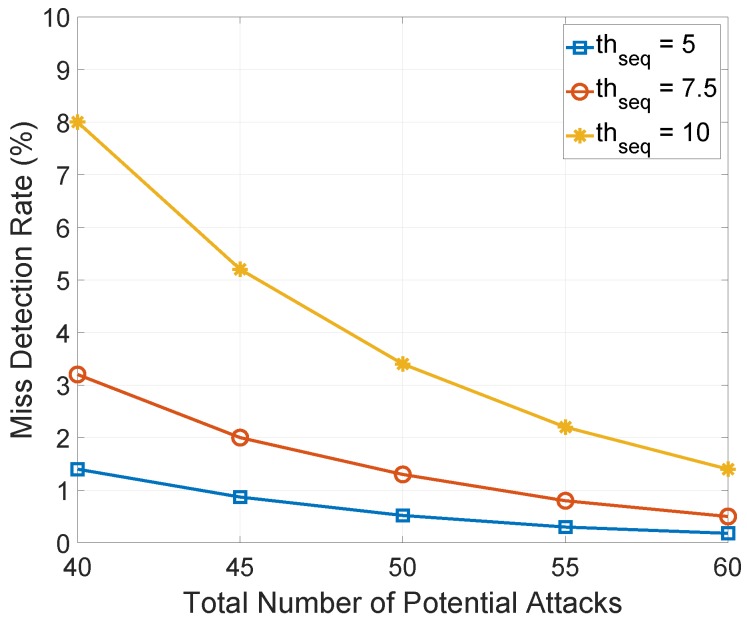
Miss detection rate against the total number of times that the malicious node decides whether to perform suppression attack.

In Figure 6, we show a numerical result of the impact of the total number of times that the malicious node decides whether to perform suppression attack $N_{atk}$ and the heuristically calculated increment threshold of sequence numbers $th_{seq}$ on the average miss detection rate based on the aforementioned analyses. Here, we assume the length of observation window is 50 s. As shown in Figure 6, when $th_{seq}$ decreases, the miss detection rate decreases significantly. This is because the malicious node broadcasts a large number of spoof data messages and causes the minimum sequence number to increase greatly, and these multicasting misbehaviors can be easily detected with a smaller $th_{seq}$. As $N_{atk}$ increases, the malicious node has more chances to perform suppression attack and multicast a series of spoof data messages within the observation window, which makes the minimum sequence number increase quickly, thus, the HED can easily compare the significant increment of a sequence number with the heuristically calculated $th_{seq}$ to detect multicasting misbehaviors.

## 5. Performance Evaluation

### 5.1. Simulation Testbed

We conduct simulation experiments using OMNeT++ [14] to evaluate the performance of the proposed approach. A 150 × 150 m${}^{2}$ square network area is considered, where 51 nodes including single source node are placed using a random uniform distribution. The communication range of each node is 30 m. To emulate low packet rate scenarios, an exponential packet injection rate with mean 0.1 packet/s is adopted and the size of each packet is 40 bytes. The radio model simulates CC2420 with a normal data rate of 250 Kbps, and 802.15.4 MAC/PHY operates with a default configuration in the 2.4 GHz band [40]. The channel error rate is set to 10%. We assume that the source node is always trusted, and 2–10% of nodes can be compromised and reprogrammed by an adversary to behave maliciously. The suppression attack rate varies between 0.0125 and 0.1 time/s, and the number of spoof data messages in each attack is 10 or 15. The total simulation time is 10,000 s. The simulation parameters are summarized in Table 1.

In this paper, we measure the performance in terms of the following four major performance metrics by altering some key simulation parameters, including suppression attack rate, number of malicious nodes, observation window (ω), number of spoof data messages ($N_{spkt}$), and filter gain constant (α). First, the detection rate is computed as the ratio of the number of detected multicasting misbehaviors to the total number of launched multicasting misbehaviors. The objective is to show the detection efficiency of the proposed scheme. Second, the packet reception rate (PRR) is calculated as the ratio of the number of received data messages to the total number of generated data messages from source node, indicating the performance resiliency of the proposed scheme. Third, a false detection rate, namely false positive rate, measures the ratio of the number of anomalous increment of sequence number due to a sudden increase of packet injection rate to the total number of detected multicasting misbehaviors. Fourth, the change of increment rate of minimum sequence number is observed and offers an explanation of how the increment rate has changed due to the multicasting misbehaviors of malicious nodes. For performance comparison, we compare the proposed HED scheme with the standard MPL multicast protocol with and without adversary, respectively.

### 5.2. Simulation Results and Analysis

**Figure 7:**
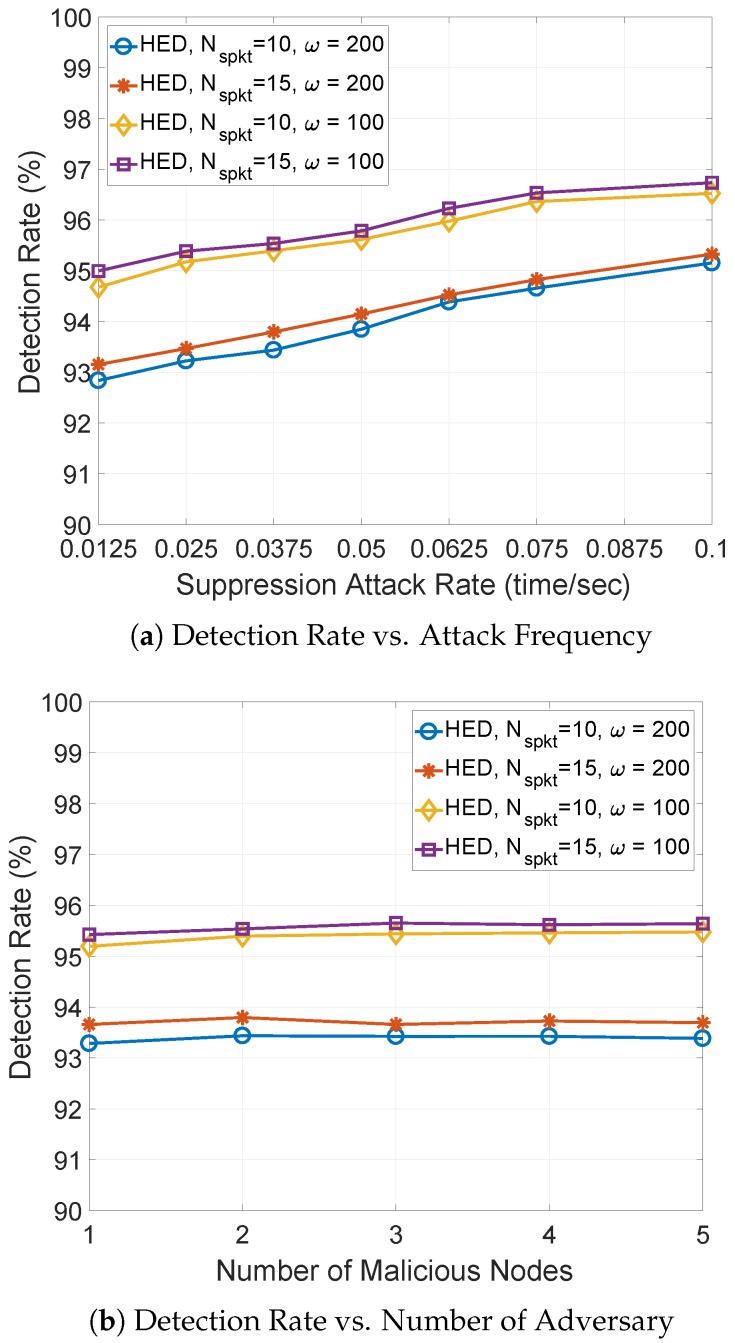
The detection rate against suppression attack rate and number of malicious nodes.

**Detection Rate:** First, we measure the detection rate against suppression attack rate, number of malicious nodes and $N_{spkt}$ in Figure 7. Overall, the detection rate of HED can be maintained above 90%. In Figure 7a, the detection rate of HED increases as the suppression attack rate increases. This is because the malicious node shows more multicasting misbehaviors with increasing suppression attack rate, however, these multicasting misbehaviors can be easily detected within adaptively adjusted observation window. The HED with larger $N_{spkt}$ achieves higher detection rate than that of the HED with smaller $N_{spkt}$. Since additional spoof data messages can cause the minimum sequence number to increase, the HED can easily compare the significant increment of a sequence number within a time period with the heuristically calculated increment threshold of the sequence number to detect multicasting misbehaviors. As the observation window reduces, a higher detection rate is achieved in comparison to a larger observation window. This is because each node can frequently compare the observed increment of a sequence number with the heuristically calculated increment threshold of sequence numbers to detect any multicasting misbehavior.

As shown in Figure 7b, the detection rate of HED is not sensitive to the number of malicious nodes. The HED is a stand-alone approach [18] where the same detection scheme is running on each node but no information is exchanged for detection. Thus, each neighbor node of malicious node can record the multicast operations of malicious node and detect the potential multicasting misbehaviors. However, the detection rate is responsive to the length of the observation window, and a higher detection rate is observed with short observation window. In this instance, the multicast operations of a malicious node can be continuously evaluated, and more multicasting misbehaviors can be detected.

**Figure 8:**
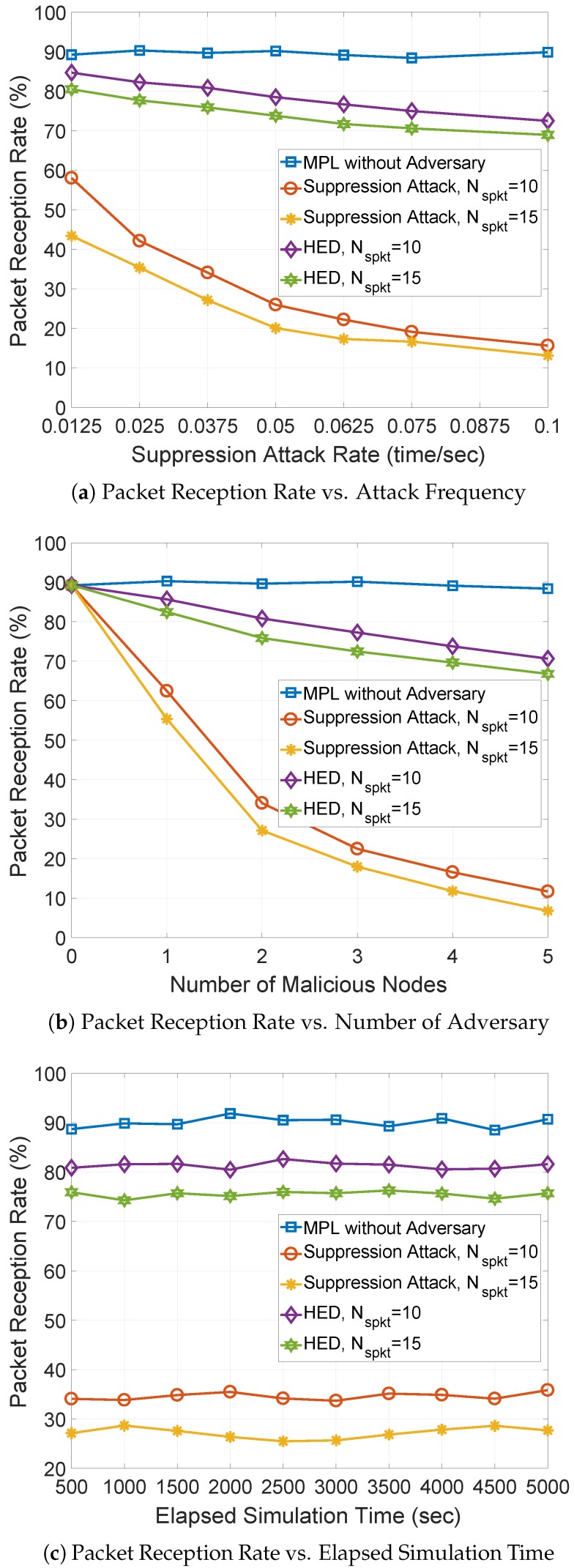
The packet reception rate (PRR) against suppression attack rate, number of malicious nodes, and elapsed simulation time.

**Packet Reception Rate (PRR):** Second, the packet reception rate (PRR) is measured against suppression attack rate, number of malicious nodes, and simulation time in Figure 8, in which the MPL without adversary provides the highest PRR, around 90%, and it is used as the upper bound of PRR. In Figure 8a, as the suppression attack rate varies between 0.0125 and 0.1 time/s, the PRR of MPL under the suppression attack with different number of spoof data messages ($N_{spkt}$) significantly decreases from 60% and 43% to approximate 15%. This is because the malicious node multicasts spoof data messages more frequently as the suppression attack rate increases, the minimum sequence number stored in the Seed Set increases more often, and less number of valid data messages from the source node will be accepted. Lower PRR is observed with larger $N_{spkt}$ = 15 under suppression attack. Since more spoof data messages make the minimum sequence number increase greatly and quickly, more valid data messages from the source node will be rejected. The HED provides lower and higher PRR than that of the MPL with and without the suppression attack, because each node records the multicast operations of neighbor nodes within an adaptively adjusted observation window to detect any anomalous increment of the sequence number. Thus, the multicasting misbehaviors of a malicious node can be easily detected, and quickly isolated from the network. The result is that more data messages can be received. As the $N_{spkt}$ increases, a lower PRR is observed. This is because more valid data messages will be rejected due to the changes in the increment of the minimum sequence number.

As shown in Figure 8b, when the number of malicious nodes increases, the PRR of MPL under suppression attack decreases. This is because more number of malicious nodes can launch more multicasting misbehaviors, and more valid data messages from source node will be rejected. However, the HED provides much higher PRR than that of MPL under the suppression attack.This is because the HED can detect the anomalous increment of sequence number due to multicasting misbehaviors of malicious node, the malicious node can be isolated and removed from the network more quickly, and more data messages can be received. In Figure 8c, as the simulation time elapses, the PRR of both MPL under the suppression attack and HED fluctuate around 77% and 30%, respectively. However, the PRR of both MPL under the suppression attack and HED are sensitive to the number of spoof data messages $N_{spkt}$, and lower PRR is observed with larger $N_{spkt}$. This is because the minimum sequence number increases greatly and quickly with larger $N_{spkt}$, and fewer data messages will be accepted.

**Figure 9:**
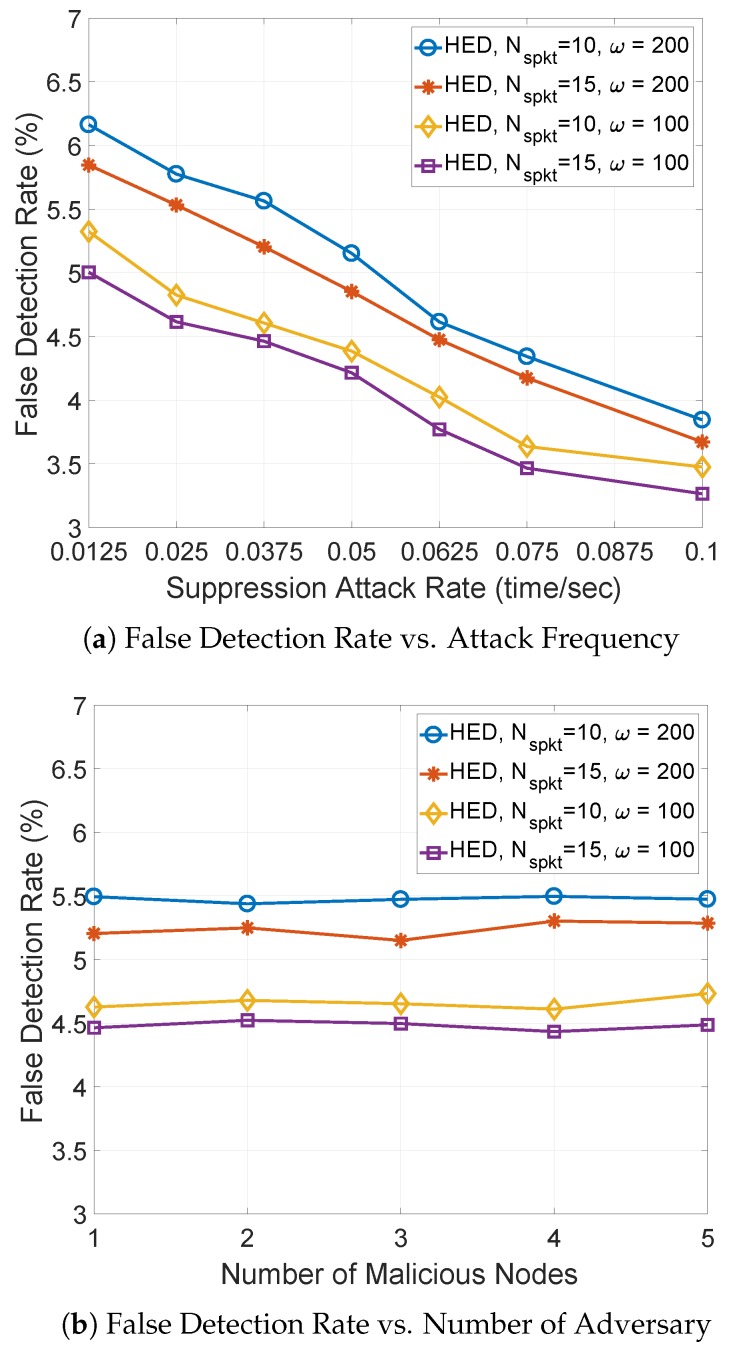
The false detection rate against suppression attack rate and number of malicious nodes.

**False Detection Rate:** Third, the false detection rate is measured by varying suppression attack rate, number of malicious nodes, and $N_{spkt}$ in Figure 9. Overall, the false detection rate of HED is below 6.5%. In Figure 9a, the false detection rate of HED decreases as the suppression attack rate increases. With larger suppression attack rate, the malicious node can show multicasting misbehaviors more frequently. However, these multicasting misbehaviors can be detected by the proposed scheme, resulting in lower false detection rate. When a short observation window is adopted, a lower false detection rate is achieved because each node can frequently compare the increment of a sequence number with threshold value to detect more multicasting misbehaviors. In Figure 9b, the false detection rate remains steady as the number of malicious nodes increases. However, the HED with smaller observation window and larger $N_{spkt}$ achieves the lowest false detection rate.

**Figure 10:**
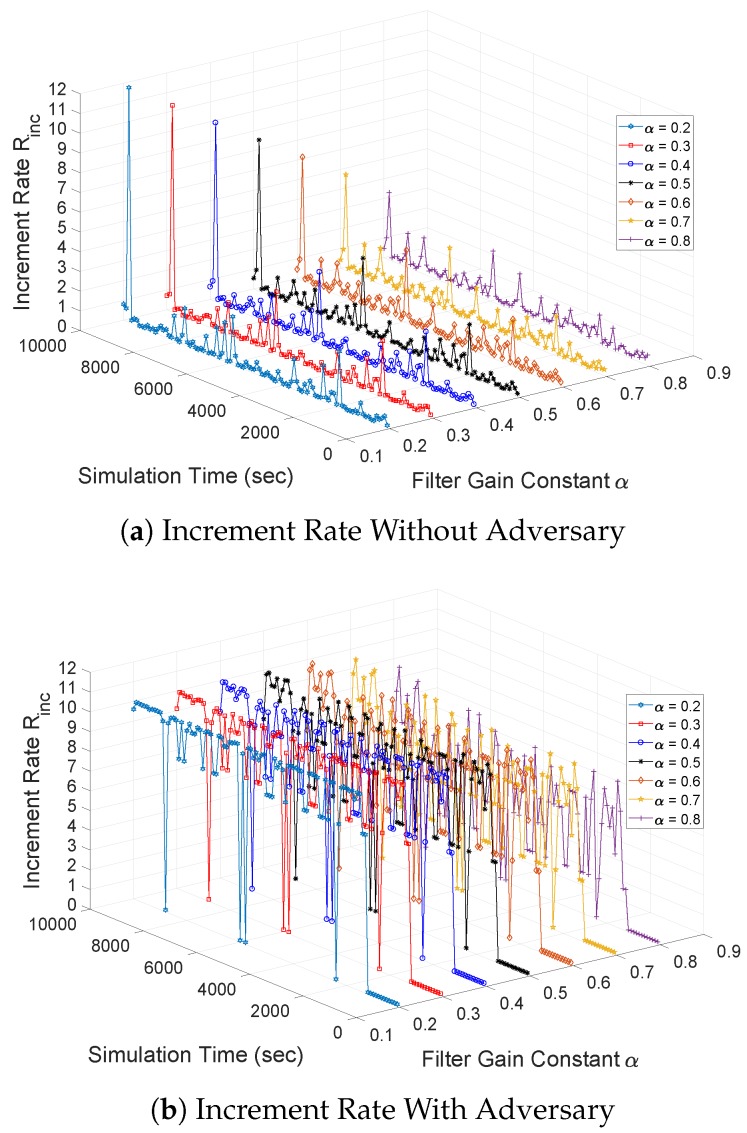
Changes of increment rate of minimum sequence number ($R_{inc}$) against simulation time and filter gain constant α.

**Changes of Increment Rate:** Finally, we measure the changes of the increment rate without and with adversary against simulation time and a filter gain constant α in Figure 10. Since a low packet rate is adopted, the increment rate of the sequence number without adversary fluctuates below 2 seq/s. Due to exponential distribution of the packet rate, it is possible to have a sudden increase of packet rate at the source node, that in turn, leads to a high increment rate. However, as shown in Figure 10a, the sudden increase of a packet rate resulting in high increment rate is very rare. Under the suppression attack, the malicious node can multicast a series of spoof data messages within a short period of time and thus, a higher increment rate is observed as shown in Figure 10b. As the value of a filter gain constant increases, the increment rate slightly decreases because the recently calculated increment rate of a sequence number contributes less weight according to Equation (1).

## 6. Discussion

In this section, we first analyze and compare the suppression attack with well-known jamming attack in terms of attack method, stealthiness, attack energy efficiency, and level of denial of service. Then, we further explore design issues and extensions of HED for future research.

The basic idea of suppression attack is that a malicious node multicasts a series of spoof data messages with continuous sequence number to increase the minimum sequence number stored in the Seed Set, which can prevent the legitimate nodes from accepting valid data messages and cause them to delete the cached data messages. For jamming attack, a malicious node needs to continually transmit a radio signal to block any legitimate access to the medium and interfere with reception. Compared to the jamming attack, the suppression attack is much stealthier. This is because the malicious node acts as a normal node but multicasts a limited number of data messages to legitimate nodes to increase the minimum sequence number stored in the Seed Set, which can cause the legitimate nodes to reject valid data messages by themselves. In addition, the suppression attack has lower energy consumption compared to that of jamming attack because the less number of attack packets (e.g., spoof data messages) are generated and multicasted by malicious node in the suppression attack. However, the jamming attack has to continually broadcast a radio signal, consuming more energy. The suppression attack makes all one-hop neighbor nodes reject valid data messages and delete the cached data messages. Thus, the valid data messages cannot be transmitted and shared further in the network. Eventually, the suppression attack can lead to an extremely severe denial of service in MPL-based LLNs.

In the HED, each node individually monitors the malicious node to detect the potential multicasting misbehaviors. Similar passive monitoring-based approaches are also found in [41,42]. Since the detection rate highly depends on how many spoof data messages are received from a single malicious node, it can be significantly reduced if multiple malicious nodes collude together. Inspired by the camouflage-based detection approach [15], in which each node pretends not to overhear on-going communication but monitors the forwarding behavior of its adjacent nodes to detect a deep lurking malicious node, we plan to extend the HED by deploying an active detection approach. The basic idea of active detection is that each legitimate node hides the information of MPL control messages, counts the number of forwarding misbehaviors, and rejects certain number of data messages from malicious node with a large number of forwarding misbehaviors.

## 7. Concluding Remarks

In this paper, we present and analyze the suppression attack with a preliminary result in MPL-based LLNs, where a malicious node multicasts a series of spoof data messages with continuous sequence numbers to prevent the normal nodes from accepting valid data messages and cause them to delete the cached data messages. To resolve this issue, we propose a heuristic-based detection scheme to efficiently detect the suppression attack in MPL-based LLNs, where each node maintains an increment rate of minimum sequence number in the Seed Set, and compares the recent increment of sequence numbers within a time period with the heuristically calculated increment threshold of sequence numbers to detect potential multicasting misbehaviors. Extensive simulation results show high detection rate and packet reception rate but low false detection rate. Thus, the proposed scheme is a viable approach against the suppression attack in MPL-based LLNs. Since radio propagation and its channel dynamics cannot easily be captured by simulation models, we plan to develop a small-scale testbed and deploy a real network in an indoor office environment to see the full potential of the proposed countermeasure.

## Figures and Tables

**Table 1:** Simulation parameters.

Parameter	Value
Network Area	150 × 150 m^2^
Number of Nodes	51
Communication Range	30 m
Packet Injection Rate	0.1 packet/s
Packet Size	40 bytes
Radio Data Rate	250 Kbps
Channel Error Rate	10%
Number of Malicious Nodes	1 to 5
Suppression Attack Rate	0.0125 to 0.1 time/s
Number of Spoof Data Messages	10 and 15
Simulation Time	10,000 s
